# Mycobacterial Infection in Recalcitrant Otomastoiditis: A Case Series and Literature Review

**DOI:** 10.3390/jcm12237279

**Published:** 2023-11-24

**Authors:** Tammy Tsai, Wei-Che Lan, Jit-Swen Mao, Yu-Chien Lee, Yung-An Tsou, Chia-Der Lin, Liang-Chun Shih, Ching-Yuan Wang

**Affiliations:** 1 Department of Otorhinolaryngology-Head and Neck Surgery, China Medical University Hospital, Taichung 404, Taiwan; d24738@mail.cmuh.org.tw (T.T.); martin_33520@hotmail.com (W.-C.L.); u106022952@cmu.edu.tw (J.-S.M.); u106022402@cmu.edu.tw (Y.-C.L.); d6638@mail.cmuh.org.tw (Y.-A.T.); 006355@tool.caaumed.org.tw (C.-D.L.); 2 Department of Audiology and Speech Pathology, Asia University, Taichung 413, Taiwan; 3 Graduate Institute of Clinical Medical Science, China Medical University, Taichung 404, Taiwan

**Keywords:** mycobacterial infections, middle ear, otitis media, clinical practice guidelines, retrospective study, literature review

## Abstract

Otomastoiditis caused by mycobacterial infections is uncommon and recalcitrant. Its clinical presentations, sometimes similar to those of common chronic suppurative otitis media, make diagnosis difficult. This retrospective study analyzed the clinical features, treatment course, and therapeutic outcomes of patients with mycobacterial otomastoiditis. The cases of six patients diagnosed with mycobacterial otomastoiditis or suspected mycobacterial infection between January 2007 and January 2019 in a single tertiary medical center in Taiwan were investigated. Information about predisposing factors, clinical features, culture reports, histopathology, treatment course, and outcomes were collected and analyzed. Relevant literature available in English was also reviewed. One patient was infected with tuberculous mycobacteria, two with suspected tuberculous mycobacteria, and three with nontuberculous mycobacteria. All six patients responded poorly to empiric antibiotic therapy, and diagnosis was not possible at their previous clinics. Five patients underwent tympanomastoidectomies; one was administered antimycobacterial medication without undergoing surgery. Mycobacterial infection was confirmed from a tissue culture or from the histopathology of the specimen, but in two patients, no definitive evidence of tuberculosis was found. Antimycobacterial medication was administered based on clinical suspicion, and improvement was noted. With appropriate therapy, all patients recovered, and no sequelae were observed after treatment. If empiric antibiotic therapy cannot achieve acceptable results, atypical infections, such as mycobacteria, should be considered. Antimycobacterial medication could be administered under clinical suspicion, serving as a diagnosis ex juvantibus. Surgical intervention might help reduce the bacterial load and obtain specimens for accurate diagnosis, but this may be unnecessary if appropriate antimycobacterial medication results in improvement. Early diagnosis and treatment can prevent complications in patients with recalcitrant otomastoiditis.

## 1. Introduction

Tuberculous otomastoiditis (TOM) is one of the rarest forms of extrapulmonary tuberculosis (TB), accounting for just 0.05–0.9% of cases, including chronic suppurative otitis media (CSOM) [1,2]. This pathogenesis of TOM may be due to secondary infection in adjacent structures, either spreading directly from the external auditory canal (EAC) with tympanic membrane perforation or spreading through aspiration via the eustachian tube. TOM can also result from a hematogenous spread from distant organs, or even from maternal infection to an infant [2,3]. TOM may manifest as painless otorrhea, multiple tympanic membrane perforations, granulations in the middle ear and mastoid, progressive conductive hearing loss, and facial nerve palsy [2]. However, these clinical symptoms are not specific, making it difficult to differentiate TOM from other conditions, such as necrotizing otitis media, cholesteatoma, otitis externa, or mastoiditis [2].

Nontuberculous mycobacteria (NTM) are commonly found in the environment, including in soil, water, plants, and animal excretions [4,5]. These bacteria can even be detected in healthy individuals. The lungs and head and neck regions are the most commonly infected areas, whereas infections in the ears are rare [6]. Patients may become infected through the EAC or the eustachian tube. Furthermore, a history of ventilation tube insertion, ear trauma, or exposure to water are also risk factors [6]. Clinical features include painless otorrhea and granulations in the EAC, middle ear cavity, and mastoid area [6]. NTM may even lead to intracranial infections [5].

The clinical presentations of otomastoiditis with mycobacterial infection are similar to those of common CSOM, but its response to empiric antimicrobial therapy is poor. Therefore, diagnosis poses a challenge and may delay appropriate treatment. To the best of our knowledge, current studies regarding the prevention of delayed diagnosis in patients with otomastoiditis caused by mycobacteria are limited. This study aimed to identify and describe cases in a single tertiary care hospital, with the goal of identifying and analyzing the differences between common CSOM and mycobacterial infections, to provide physicians with a better method for early diagnosis and treatment.

## 2. Materials and Methods

### Study Design

With the approval of the China Medical University Hospital Institutional Review Board, we retrospectively reviewed the medical charts of patients admitted for otomastoiditis with mycobacterial infection at a single tertiary hospital between January 2007 and January 2019. Ultimately, six patients between 40 and 66 years of age met the inclusion criteria, including those with a diagnosis confirmed using tissue culture samples and pathology reports, or those with suspected otomastoiditis with mycobacterial infection based on clinical improvement after administration of antimycobacterial medication. Among these patients, one had a TB infection, two had suspected TB infections, and three had NTM infections. Data were analyzed and discussed, including age, sex, pathogen, past medical history, predisposing factors, clinical features, complications, imaging, culture reports, histopathology, time of operation, time to diagnosis, whether the treatment course included antibiotics, and the sequelae after treatment.

## 3. Case Presentations

### 3.1. Case 1

A 59-year-old female with valvular heart disease had persistent left postauricular pain for more than 1 year. She was previously treated with topical antibiotic ear drops at a hospital. Her response to treatment was poor; therefore, she visited our clinic for help in September 2007. At this visit, a 50% central perforation of the left tympanic membrane with a mucoid discharge was noted upon physical examination. Pure-tone audiometry demonstrated a severe mixed type of hearing loss in the left ear. A case of chronic otitis media refractory to empirical antimicrobial therapy was suspected, and type I tympanoplasty via a post-aural approach was performed. Granulated tissue in the middle ear cavity was noted during surgery, and histopathology revealed acute and chronic inflammation.

**Figure 1:**
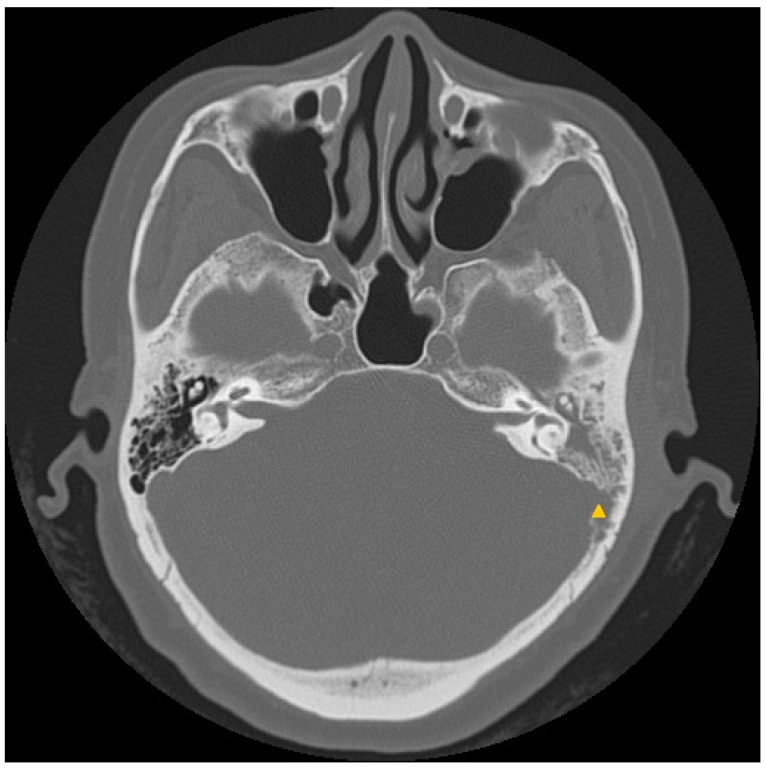
Case 1: A high-resolution computed tomography scan of the temporal bone shows erosion of the sigmoid plate (▲) with opacification of the left mastoid antrum, mastoid air cells, and middle ear cavity.

Four months after the procedure was performed, purulent otorrhea continued to recur and the patient’s response to medication was poor. High-resolution computed tomography (HRCT) of the temporal bones revealed erosion of the sigmoid plate with opacification of the left mastoid antrum, mastoid air cells, and middle ear cavity (Figure 1). The patient underwent mastoidectomy, which revealed granulation tissue in the mastoid air cells and the EAC. A tissue specimen was sent for bacteriological analysis, and an NTM infection was confirmed, while the culture smear was positive for acid-fast bacilli. Deoxyribonucleic acid sequence amplification using polymerase chain reaction (PCR) was performed and *Mycobacterium abscessus* was identified. Accordingly, a 6-month course of moxifloxacin and clarithromycin was prescribed. The postauricular wound showed obvious improvement and healed 6 months after the third and last surgical procedure.

### 3.2. Case 2

**Figure 2:**
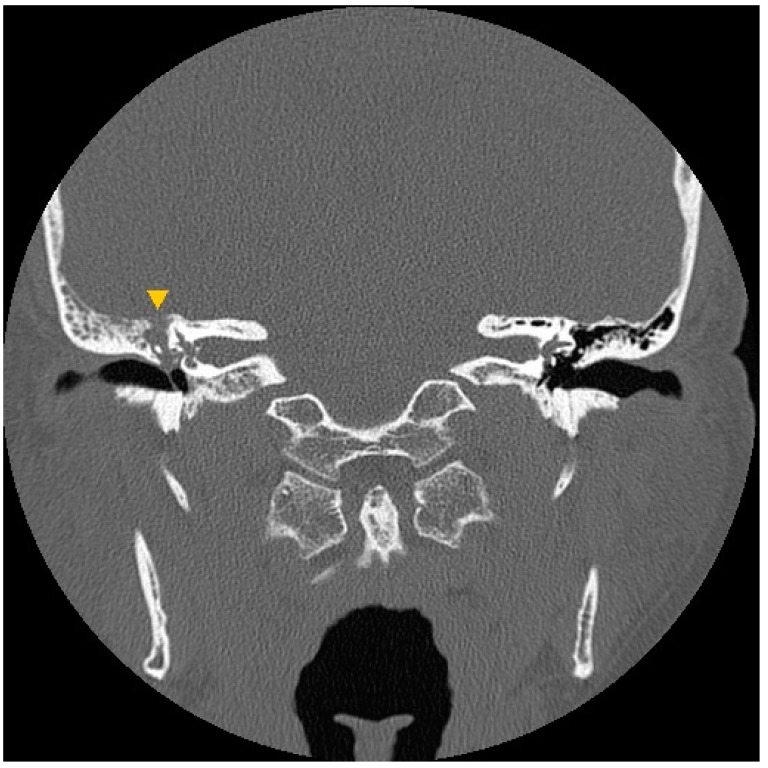
Case 2: A high-resolution computed tomography image of the temporal bone shows obliteration of the right mastoid air cells and middle ear cavity, with erosion of the right tegmen (▼).

**Figure 3:**
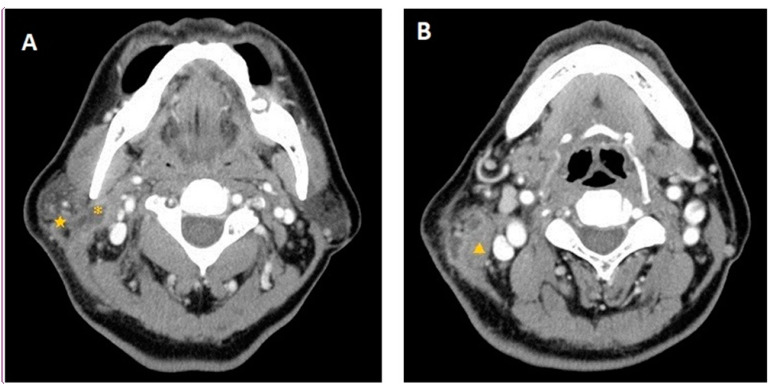
Case 2: Computed tomography images of the neck show (**A**) focal fluid accumulation in the right parapharyngeal (*) and right parotid (★) spaces; and (**B**) right sternocleidomastoid muscle swelling (▲).

A 66-year-old male with a history of lung adenocarcinoma presented to our clinic in December 2008 with a 2-week history of painful swelling in the right infra-auricular region and right otalgia. Physical examination revealed a tender right infra-auricular mass measuring approximately 3 × 3 cm^2^. Otoscopy revealed a 30% central perforation of the right TM with massive purulent discharge and considerable granulation tissue over the mesotympanum. HRCT of the temporal bones revealed opacification of the right mastoid air cells and middle ear cavity, with erosion of the tegmen tympani (Figure 2). Computed tomography of the neck revealed focal fluid accumulation in the right parapharyngeal and right parotid spaces (Figure 3A). Right-sided sternocleidomastoid muscle swelling was also observed (Figure 3B). The patient was diagnosed with right mastoiditis and possible parotitis. He first underwent a right mastoidectomy with needle aspiration of the right upper neck abscess, followed by incision and drainage of the right upper neck 2 days later due to persistent swelling with suppurative discharge. Thereafter, three episodes of debridement of the right neck and post-aural wound were performed because of recurrences and poor wound healing with dehiscence and granulation. The right mastoidectomy and post-aural wound pathology revealed granulation tissue with chronic and acute inflammatory cell infiltration. Culture reports showed no positive findings. As a result of the refractory condition following standard antimicrobial therapy and surgery, the patient was treated with trimethoprim–sulfamethoxazole, rifampin–isoniazid, ethambutol, and pyrazinamide for 6 months for suspected TB otomastoiditis and Bezold’s abscess. This treatment course was recommended by infectious disease specialists. Following completion of the treatment regimen, the only remaining abnormality was an atrophic scar involving the TM. The patient remained disease-free after 12 months of follow-up.

### 3.3. Case 3

A 61-year-old female with diabetes mellitus visited our otolaryngology department in May 2013 with a chief complaint of left otorrhea and otalgia for 1 year after swimming. Previously, she was treated with topical antibiotic ear drops at a hospital, but the response to the medication was poor. Two perforations in the left tympanic membrane were observed on physical examination at our department. Pure-tone audiometry showed a 63 dB mixed type of hearing loss on the left side with an air-bone gap of approximately 35 dB and a type B tympanogram. Left tympanoplasty with modified radical mastoidectomy was performed for left chronic otitis media. Histopathological examination revealed granulomatous inflammation with caseation. Based on a histopathological diagnosis, the patient was treated with rifampin-isoniazid, ethambutol, and pyrazinamide for 9 months. The symptoms subsided, and the patient’s condition gradually improved.

### 3.4. Case 4

**Figure 4:**
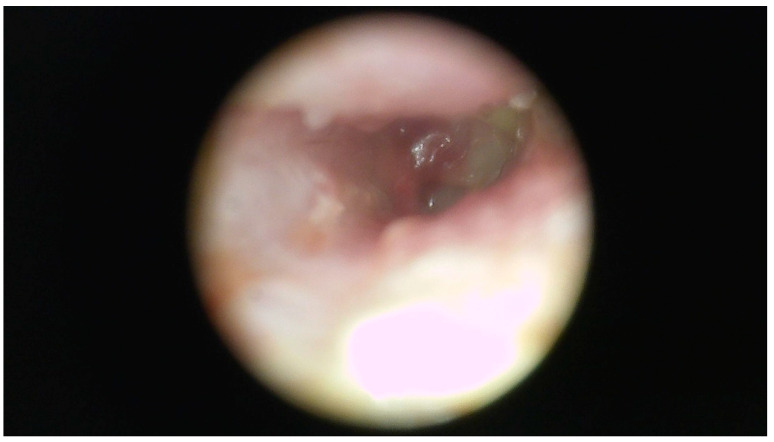
Case 4: An endoscopic image of the right external ear canal shows extensive pus and keratin debris, with swelling of the canal wall.

**Figure 5:**
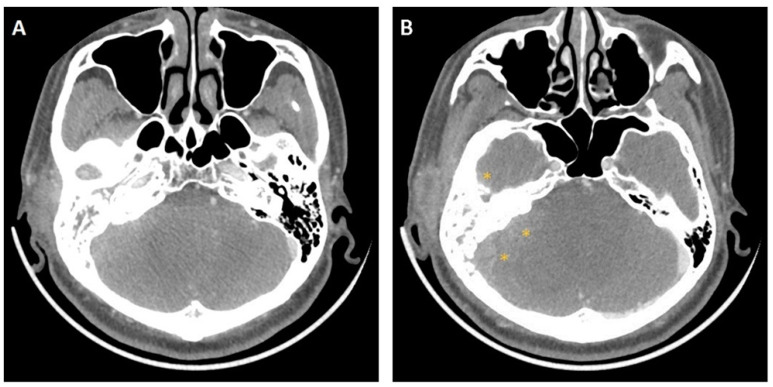
Case 4: High-resolution computed tomography scans show (**A**) infiltration of the soft tissue, with increased enhancement in the right mastoid and middle ear, and (**B**) intracranial extension to the right lateral areas of the posterior and middle cranial fossae (*).

A 49-year-old male without a specific systemic disease presented in November 2013 with a 3-month history of right otorrhea, intermittent otalgia, and progressive hearing impairment. An unsteady gait was also noted. Otoscopy revealed pus and keratin debris on the right EAC (Figure 4). Empiric antimicrobial therapy and ofloxacin otic solution were administered, but with poor response. Contrast-enhanced HRCT and brain magnetic resonance imaging showed soft-tissue infiltration in the right mastoid cells, tympanic cavity, petrous part of the temporal bone, EAC, temporal scalp, and cerebellar hemisphere (Figure 5A,B). Temporal bone destruction and intracranial extension with lesions on the dura were also found, suggesting a case of skull base osteomyelitis. Debris in the right EAC was obtained from the patient’s previous hospital and sent for histopathological study, which only showed acute and chronic inflammation. Pus culture revealed no specific findings. However, the otorrhea persisted. After being briefed by infectious disease specialists, the patient agreed to be initially treated for NTM infection with rifampin, ethambutol, and azithromycin for 1 week. The diagnosis was then revised to TB infection, and the patient was treated with an antibiotic regimen of rifampin–isoniazid, ethambutol, and pyrazinamide due to poor response to the initial regimen. After 12 months of treatment, the patient was disease-free, and brain magnetic resonance imaging did not show recurrence of the disease.

### 3.5. Case 5

**Figure 6:**
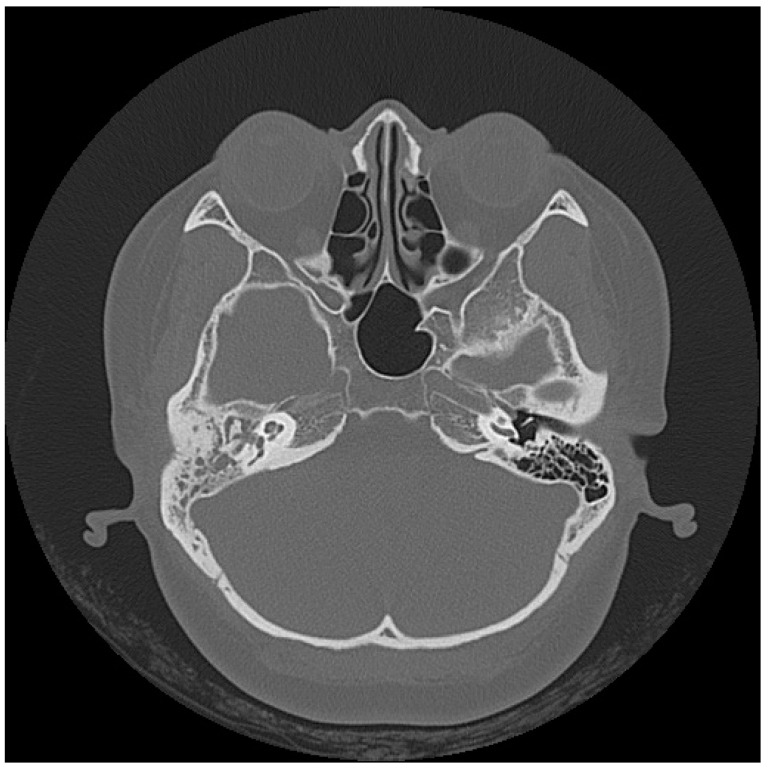
Case 5: A high-resolution computed tomography scan of the temporal bones shows diffuse opacification of the right mastoid air cells and middle ear cavity.

A 40-year-old female without any specific systemic disease presented in July 2017 with intermittent right otorrhea for 10 months. She initially visited a local clinic, where she received antibiotic treatment, but the disease showed no positive response to the medication. Hence, she visited our otorhinolaryngology department, where otoscopy revealed a right bulging dull tympanic membrane and swelling of the right EAC with congestion. She was first treated with a ventilation tube to treat suspected right acute otitis media and acute otitis externa. However, the patient showed no improvement, and swelling of the right EAC with congestion was still noted during the follow-up. In addition, knocking pain over the mastoid process was also observed. HRCT of the temporal bone showed diffuse opacification of the right middle ear cavity and mastoid air space (Figure 6). Multiple areas of bone destruction with defects in the lateral and inferior walls were also noted, suggesting skull base osteomyelitis.

Right chronic otitis media was suspected and thus right tympanoplasty with mastoidectomy was performed. The ventilation tube fell out and no other foreign bodies were found during the operation. Bacteriological analysis showed NTM infection, and the culture smear was positive for acid-fast bacilli. Deoxyribonucleic acid sequence amplification by PCR was performed, and the presence of *M. farcinogenes* was determined. The patient was treated with rifampin–isoniazid, ethambutol, and clarithromycin for NTM-related otitis. Nine months after completing treatment for NTM, a follow-up computed tomography scan showed a thickened right tympanic membrane with a clear middle ear and mastoid cavity.

### 3.6. Case 6

**Figure 7:**
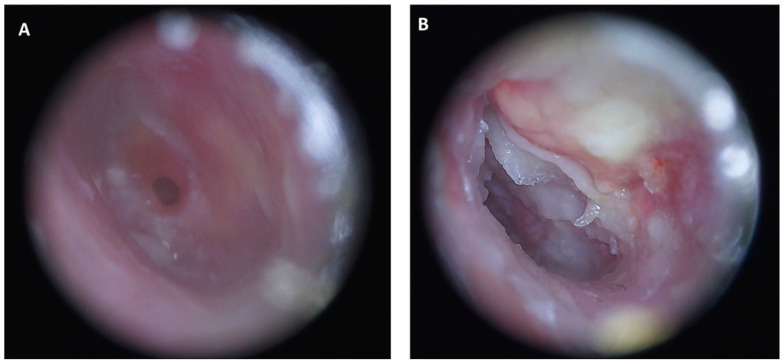
Case 6: (**A**) An infected left external auditory canal and small perforation on the left tympanic membrane. (**B**) Exudate in the left external auditory canal was still found during follow-up after type I tympanoplasty with atticoantromastoidectomy was performed.

In August 2018, a 58-year-old female with gastroesophageal reflux disease visited our otorhinolaryngology department with intermittent left otalgia, aural fullness, otorrhea, and tinnitus of 2 years. She visited a local clinic, which found tympanic membrane perforation and treated her for chronic otitis media with empiric antibiotics and topical otic solution. However, the symptoms recurred. She was then referred to our department, where an infected left EAC and a small perforation of the left tympanic membrane were noted on physical examination (Figure 7A). The chronic otitis media infection showed poor response to empiric antibiotic therapy; therefore, type I tympanoplasty with atticoantromastoidectomy was performed. Histopathology revealed only granulomatous inflammation. Poor postoperative wound healing was noted, with postauricular erythematous swelling, wound dehiscence, and some purulent discharge, all of which were refractory to empiric antibiotic therapy. Dehiscence with granulation at the left postauricular wound, granulation at the tympanic membrane, and exudate in the left EAC were also observed during the follow-up (Figure 7B). Incision and debridement were performed on the post-aural infectious wounds. NTM was found in the pus culture from the left EAC, and culture smear was positive for acid-fast bacilli. *M. abscessus* was identified using PCR. Rifampin–isoniazid, ethambutol, and pyrazinamide were prescribed. Nine months after completing treatment for NTM, the mesotympanum was clear, and no recurrent infection was noted on the subsequent HRCT scan.

## 4. Results

Our study included two male and four female patients (Table 1). Three of the six patients had previous ear disease or predisposing factors: Case 1 had CSOM and previously underwent tympanoplasty; Case 5 underwent ventilation tube insertion before the occurrence of recalcitrant otomastoiditism, which promotes the risk of biofilm formation by mycobacteria, leading to infection in the ear [7]; and Case 3 had diabetes mellitus, which is considered to be an immunosuppressive condition and recognized as a risk factor for TB and NTM infection [8].

Four patients had an initial presentation of tympanic membrane perforation (Cases 1, 2, 3, and 6): three patients had a central perforation (Cases 1, 2, and 6) and one patient had multiple perforations (Case 3). All the patients in this study had mastoiditis. Among them, one patient (Case 2) had Bezold’s abscess, and two patients (Cases 4 and 5) had skull base osteomyelitis. Involvement of the right cerebellar hemisphere was implicated in Case 4.

Cultures were positive in three cases, all of which were NTM infections. Using PCR, *M. abscessus* was identified in Cases 1 and 6 and *M. farcinogenes* was identified in Case 5 (Table 2). Pathology specimens were available for all cases. In most pathology reports, we observed granulation tissue with inflammation. Granulomatous inflammation with caseation was the most specific finding for TOM, which was found in one patient (Case 3).

The average time from the first visit to diagnosis was 3 months (range 1–7 months) and the average length of the treatment course was 8.5 months (range 6–12 months; Table 2). Most patients underwent several surgeries, such as tympanoplasty, mastoidectomy, or debridement, while one patient (Case 4) did not undergo surgery. Poor healing with dehiscence and granulation at the postauricular wound was noted in two patients after surgery (Cases 2 and 6). After appropriate antibiotics, the patients no longer experienced otorrhea, tympanic membrane perforation, or major permanent sequelae such as peripheral facial palsy, postauricular fistulae, labyrinthitis, meningitis, osteomyelitis, or subperiosteal, cerebral, or cerebellar abscesses [9].

## 5. Discussion

Mycobacterial otomastoiditis is rare and refractory to standard antibiotic treatment. It often mimics other common CSOMs, necrotizing otitis media, cholesteatoma, or otitis externa, making its diagnosis difficult [10]. TM perforations can be observed in affected patients, but this condition varies and either single or multiple perforations may be observed [11,12,13]. In our study, only one patient had multiple tympanic membrane perforations. Often, delay in diagnosis and proper management may lead to serious complications involving the auditory and central nervous systems [12]. In the present study, in addition to mastoiditis, other complications included Bezold’s abscess and skull base osteomyelitis with cerebellar hemisphere involvement, which were noted in different patients.

The diagnosis of TOM should strongly rely on clinical conditions and should be considered in patients with chronic ear infections with a known or suspected tuberculous infection at other sites in the body [13,14]. Similar to other extrapulmonary forms, tuberculous lesions of the ear usually present with low bacillus concentrations [15], and with the use of aminoglycoside ear drops, the concentration may be even lower [16]. Therefore, microbiological reports may be unreliable and delay treatment. However, suspected TOM without clinical findings should not be excluded, even in cases where no evidence of tuberculosis is present [1,14]. The treatment of TOM should be discussed with patients, and a diagnosis ex juvantibus should be considered [1], as reported by Dardel et al. in 2001 [17]. In our study, although culture, PCR, and histopathology showed no definite evidence of tuberculosis in Cases 2 and 4 (Table 2), TOM was suspected based on the clinical features; the patients’ conditions significantly improved after administering antibiotics for TOM.

Surgical biopsy to obtain diseased tissue and pus for pathology and culture may be necessary for diagnosis. However, eradication surgery is rarely required when appropriate antituberculosis medication is administered as such medication can usually treat the disease effectively [14,18]. The primary role of surgery is to expedite the diagnosis and ensure its accuracy to prevent delayed therapy and possible complications [19]. However, if a patient undergoes tympanoplasty or mastoidectomy without appropriate drug therapy, the condition might become complicated with fistulae, non-healing of suture lines, or tympanoplasty failure [19]. In our study, poor healing at post-aural wounds with granulation and dehiscence was noted in two patients after surgeries (Cases 2 and 6). In both cases, we cultured samples from the dehiscent wounds, which finally improved after the appropriate antibiotic treatment. Serious sequelae, such as subperiosteal abscess and removal of the sequestrum, may still need surgical intervention [20,21]. Furthermore, treating facial nerve palsy through surgery remains controversial because the prognosis of facial palsy depends on early diagnosis and treatment with antituberculosis drugs rather than decompression [20,21].

According to Flint et al. (1999), the ideal treatment for otomastoiditis caused by NTM infection is the complete excision of the affected tissue [4]. Unlike TOM, otomastoiditis due to NTM infection has variable and usually limited sensitivity to antibiotics [4]. Thus, surgery combined with antibiotics is required to treat such infections [4]. However, in 2015, Lundman et al. reported 16 cases of otomastoiditis caused by NTM and found three patients who did not undergo any surgery for treatment [5]. These patients were diagnosed by culturing polyps found in the EAC; healing was rapid, and medication included only antibiotics [5]. In the study of Lundman et al., the aim of the operation was to diminish the local bacterial load while causing as little damage as possible [5]. In our study, three patients with NTM otomastoiditis (Cases 1, 5, and 6) underwent surgery, but were mainly healed via a complete antibiotic regimen.

The low incidence of mycobacterial infection in otomastoiditis and difficulty in diagnosis were limitations that resulted in the small number of patients enrolled in this study. Despite this limitation, we made a diagnosis ex juvantibus of the infection in the patients without definite proof of *Mycobacterium* infection but with significant clinical improvement after tentative antimycobacterial treatment. According to recent literature, in patients with *M. abscessus* otomastoiditis, quality of life may temporarily be poor due to treatment. Hearing loss with adverse events such as nausea and vomiting may be noted after antibiotic treatment or surgery [22]. In the cases investigated in our study, no obvious sequelae were observed during long-term follow-up after proper management.

**Figure 8:**
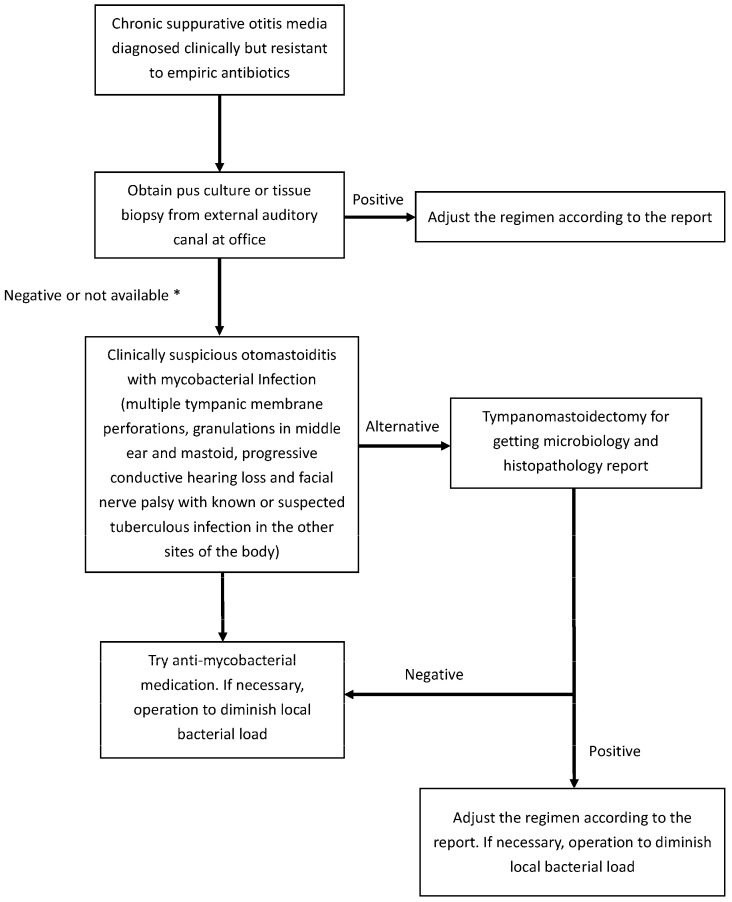
An algorithm for diagnosing and managing recalcitrant otomastoiditis in which mycobacterial infection should also be considered. * Differential diagnoses such as malignant or benign tumor or other inflammatory or infectious diseases should be ruled out via pus culture and tissue biopsy.

Mycobacteria have developed resistance to most conventional antibiotics; therefore, treatment for this pathogen is usually unsatisfactory, and increases in the number of infections have been observed [23,24]. To treat patients with otomastoiditis caused by *M. abscessus*, a multidrug regimen administered for a minimum of 6 months should be required. Moreover, the symptoms of otalgia might imply local disease spread, predicting a significantly prolonged antimycobacterial treatment duration [7]. In our study, two patients had otomastoiditis caused by *M. abscessus* (Cases 1 and 6), and a longer treatment course of 9 months was noted for the patient described in Case 6 who had otalgia. The medical regimen was difficult to standardize; however, neither otorrhea nor tympanic membrane perforation was noted in any of our cases after administering the appropriate antibiotics recommended by infectious disease specialists. Medical treatment included rifampin–isoniazid, ethambutol, and pyrazinamide for TOM and moxifloxacin, clarithromycin, rifampin–isoniazid, ethambutol, and pyrazinamide for NTM otomastoiditis. Specialist consultation is important because each treatment course has both effectivity and adverse effects that need to be evaluated carefully [7]. The purpose of surgical treatment was not to completely eradicate the infected tissue, but to decrease the bacterial load and obtain specimens for diagnosis using histology or tissue culture; thus, appropriate antibiotics should be administered to treat mycobacterial infections. Furthermore, mycobacterial infection should be strongly suspected in cases of chronic otitis media of unknown origin and granulation tissue in the EAC or middle ear when the otitis is resistant to standard antimicrobial therapy. We suggest an algorithm for diagnosing and managing recalcitrant otomastoiditis, in which mycobacterial infection should also be considered (Figure 8).

## 6. Conclusions

Diagnosing otomastoiditis with mycobacterial infection has always been challenging. Mycobacteria should be considered if empiric antibiotic treatment does not achieve a response. In addition, in cases without specific findings in the tissue culture or histopathology, antimycobacterial medication based on clinical suspicion may be administered. Surgical intervention could be helpful in obtaining specimens for diagnosis or reducing bacterial load. Early diagnosis and appropriate treatment regimens are key to eradicating mycobacterial otomastoiditis and preventing morbidity.

## Figures and Tables

**Table 1:** Patient and symptomatic characteristics.

Case	Age (years)/Sex	Pathogen	Past History/Predisposing Factors	Clinical Features/Complications
1	59/F	NTM ^a^	Heart disease CSOM undergoing tympanoplasty	Mastoid cells abscess with granulation tissue
2	66/M	TB ^b^	Lung cancer	Bezold’s abscess
3	61/F	TB ^c^	DM	Mastoiditis, granulation
4	49/M	TB ^b^	None	Skull base osteomyelitis Right mastoid and cerebellar hemisphere involvement
5	40/F	NTM ^a^	VT insertion	Mastoiditis Skull base osteomyelitis
6	58/F	NTM ^a^	None	Poor wound healing after tympanoplasty with mastoidectomy

^a^ Diagnosed via culture and PCR; ^b^ Diagnosis was confirmed via good response to antituberculosis medication; ^c^ Diagnosed via caseating granuloma in pathology. CSOM, chronic suppurative otitis media; DM, diabetes mellitus; F, female; M, male; NTM, nontuberculous mycobacteria; TB, tuberculosis; VT, ventilation tube.

**Table 2:** Microbiology, histopathology, clinical course, and sequelae.

Case	Tissue Culture	PCR	Histopathology	Number of Operations	Poor Wound Healing	Time to Diagnosis (Months)	Treatment Course (Months)	Cured?	Sequelae
1	Positive	*M. abscessus*	Granulation tissue Lymphocytic infiltration	4	-	7	6	Yes	-
2	Negative	-	Granulation tissue with chronic and acute inflammatory cell infiltration	4	+	4	6	Yes	-
3	Negative	-	Granulomatous inflammation, caseating	2	-	1	9	Yes	-
4	Negative	-	Chronic and acute inflammatory cell infiltration	0	-	1	12	Yes	-
5	Positive	*M. farcinogenes*	Granulation tissue	1	-	1	9	Yes	-
6	Positive	*M. abscessus*	Granulomatous inflammation	2	+	4	9	Yes	-

PCR, polymerase chain reaction.

## Data Availability

The data presented in this study are available on request from the corresponding author. The data are not publicly available due to privacy and ethical restrictions.

## Abbreviations

CSOM	Chronic suppurative otitis media
EAC	External auditory canal
HRCT	High-resolution computed tomography
NTM	Nontuberculous mycobacteria
PCR	Polymerase chain reaction
TB	Tuberculosis
TOM	Tuberculous otomastoiditis
